# Powered stapling system with gripping surface technology for pulmonary resection of lung cancer: real-world clinical effectiveness

**DOI:** 10.1186/s12962-022-00398-5

**Published:** 2022-12-23

**Authors:** Chongzhi Gan, Fuchun Zeng, Wei Cong, Tiange Tang, Gang Feng

**Affiliations:** 1 Department of Thoracic Surgery, Sichuan Provincial People’s Hospital, Chengdu, 610072 Sichuan People’s Republic of China; 2 Department of Health Policy and Management, School of Public Health and Tropical Medicine, Tulane University, 1440 Canal St, New Orleans, LA USA

**Keywords:** Manual staplers, Powered staplers, Gripping surface technology, Pulmonary resection, Lobectomy, Segmentectomy, NEOVEIL

## Abstract

**Objectives:**

Surgical lung resection involves a critical task of stapled ligation and transection of major vascular structures and tissue, which may lead to bleeding and complications. A newer powered stapling system with Gripping Surface Technology (GST) was introduced to account for tissue movements. This study aimed to examine the real-world effectiveness of GST system on intraoperative and postoperative outcomes of pulmonary resection.

**Methods:**

A retrospective analysis was conducted using the electronic medical records of Sichuan Provincial People’s Hospital between July 2020 and March 2021 in China. Patients who underwent their first procedures of single-port lobectomy or multi-port segmentectomy by video-assisted thoracoscopic surgery were identified and grouped as GST group or manual stapler group (manual group) by the stapler types. The intraoperative outcomes such as bleeding rate, blood loss volume, and intervention rate at the staple line (including intraoperative pressure, suture, and electrocoagulation) were documented by trained nurses during the surgery. Propensity score matching was performed between the two groups, controlling forage, BMI, smoking history, history of surgery, complications, and level of complexity of pneumonectomy.

**Results:**

A total of 108 matched patients were included in the analysis (54 in the GST group and 54 in the manual group). GST group had lower risks for intraoperative bleeding (22.8% vs 51.9%; p = 0.003) and intraoperative interventions (31.5% vs 55.6%; p = 0.02), compared to the manual group. A decrease in the intraoperative blood loss was observed in the GST group, but not statistically significant (134.39 ± 52.82 ml vs 158.11 ± 73.14 ml, p = 0.102). The use of NEOVEIL (reinforcement material to prevent air leakage from the staple line) intraoperatively during surgery was significantly lower in the GST group (24.1%) than in the manual group (50%, p = 0.01).

**Conclusion:**

The GST system was associated with better intraoperative outcomes in clinical practice in China.

## Introduction

Lung cancer is one of the biggest public health concerns worldwide as it is the leading cause of global cancer deaths in both men and women and ranked the second in the cause of global incident cancer cases [1]. In China, lung cancer is the most prevalent cancer and the first leading cause of cancer death, with an estimation of 733,000 incident cases and 610,000 lung cancer deaths each year, posing a heavy burden on both Chinese society and families [2, 3]. It is estimated that the economic burden cost by lung cancer would be $40.4 billion by the time of 2025 [4].

In recent years, video-assisted thoracoscopic surgery (VATS) has become the mainstream approach for pulmonary resection [5]. The most common perioperative complications after VATS include intraoperative bleeding, acute myocardial ischemia, acute respiratory distress syndrome (ARDS), prolonged air leak, and pneumonia [6–8]. Additionally, leaking or oozing can obscure the physicians’ vision and hence result in unfavorable clinical outcomes [9]. Furthermore, the limited operation space significantly increases the difficulty of manual suturing and makes surgical stapler essential for bronchial stump closure and pulmonary hilum vessel ligation in VATS [10].

Surgical staplers are widely used to assist surgeons to reach better clinical outcomes because surgical staplers can reduce operation time, tissue trauma, and surgical contamination, compared to manual operations. Although manual staplers used to be the standard tool in VATS, powered staplers were invented for easier operation and further reducing the risks of air leak and bleeding through even nail formation force [11, 12]. Powered staplers also demonstrate better clinical and economic performance than manual staplers. The Miller et al. study reported that the use of powered staplers is associated with a significantly shorter hospital length of stay, lower total hospital costs (as well as room and board cost), lower rates of the composite hemostasis complications, and less transfusion compared to manual staplers [13]. The use of powered staplers is also associated with significantly lower adjusted total hospital cost, adjusted supply cost, and adjusted operating room cost compared with the use of manual staplers [14, 15].

A new generation stapling system with the gripping surface technology (GST) is designed to account for tissue movements associated with the viscoelastic response of the tissue [16] and provide a superior tissue gripping force without causing additional trauma [17]. The GST system is found to be associated with better operative outcomes, including less intraoperative bleeding and intraoperative interventions in both pre-clinical and clinical settings [17, 18]. Thus, this study aimed to investigate the real-world effectiveness of the GST system on the intraoperative and postoperative clinical outcomes of VATS in the Chinese hospital setting.

## Materials and method

### Data source

The demographic profile, socioeconomic status, clinical characteristics, laboratory results, operative reports, and hospitalization costs were extracted from the EHR system of SPPH. Intraoperative outcomes including intraoperative bleeding and intervention were documented by trained nurses during the surgery. Physicians at the study center responsible for data collection were trained to monitor and process the collected data to assure that all the data were accurately documented. Baseline variables included demographic and operation-related clinical characteristics. Demographic variables included age and gender. Clinical variables included body mass index (BMI), smoking history, operation history, comorbidity, level of complexity of pneumonectomy based on clinical knowledge and experience, cancer type, tumor location, access type, pulmonary fissures, pulmonary functions, and pleural adhesions.

### Study cohort

A retrospective analysis was conducted using the electronic health records (EHR) of Sichuan Provincial People’s Hospital (SPPH) between July 2020 and March 2021 in China. The eligible participants were the patients with lung cancer who were admitted to SPPH between July 1, 2020, and March 31, 2021, and underwent their first procedures of single-port lobectomy or multi-port segmentectomy by VATS using either the manual staplers or the staplers powered by GST system. Patient surgical dates were defined as the index dates. The baseline period was defined as the time period between July 1, 2020, and March 31, 2021. The GST system was composed of Echelon Flex GST powered staplers and GST cartridges while the manual stapler group included domestic-branded manual staplers and their cartridges. The eligible participants were also required to be at least 18 years old at admission. Patients who had a complete pulmonary fissure, severe pleural adhesions, or poor pulmonary functions were excluded from the sample. Patients who underwent multiple segments or multiple lobe resections were also excluded from the study. All the eligible participants were divided into the GST or manual group based on whether the GST system was used in their surgeries.

### Surgical procedures

Pulmonary resection was performed while patients were under general anesthesia and double-lumen endotracheal tubes were used for ventilation during the surgery. VATS lobectomy was performed via the single port method and VATS segmentectomy was performed via the multi-port method; both procedures were taken under monitor vision only.

After completing the procedures for lobectomy or segmentectomy, a sealing test was conducted before wound closure. The sealing test was performed by reinflating the lung with water on the wounded side to determine whether there was any air leakage. If any air leakage was detected, fibrin glue and a PGA sheet (NEOVEIL sheet) were used to cover the surface of leaked pulmonary tissue, otherwise, the leaked part was sutured by non-damaging thread. Chest X-ray was performed on the first day after operation to confirm the inflation of the lung on the surgical side. Chest drainage tube was removed if the lung expansion was good, chest drain was less than 300 ml, and no air leakage on the tube.

### Stapler operation

In lobectomy, staplers were used to sever the blood vessels, bronchi, and the lung parenchyma. In segmentectomy, staplers were used to sever the bronchi and the lung parenchyma. For larger vessels such as basilar artery, staplers were applied.

### Outcomes

The primary outcomes included all the bleeding-related events: intraoperative bleeding rate and volume, and intraoperative intervention rate. Intraoperative bleeding was defined as bleeding from the incisions and bleeding from the lung parenchyma, bronchial artery or vessels after endo-staplers were applied. Intraoperative intervention was defined as intraoperative bleeding controlled using suture repair, compression, or coagulation hemostasis. The secondary outcomes included both intraoperative and postoperative outcomes. The secondary intraoperative outcomes included the NEOVEIL usage (reinforcement material to prevent air leakage from the staple line), intraoperative air leakage (no bubble, countable bubble, and continuous bubble), and the operation time. The secondary postoperative outcomes included drainage tube placement longer than 5 days, conversion to open surgery, reoperation during the admission, and length of stay.

### Statistical analyses

The statistics in mean, standard deviation (SD), numbers, and proportion in percentage were used to report all the quantitative variables where appropriate. Mean and standard deviation were used for descriptive analysis of continuous variables and frequency and proportion in percentile were used for descriptive analysis of categorical variables. T-test and Wilcoxon rank sum test were used to compare the continuous variables of GST and manual group. Chi-square test and Fisher exact test were used to compare the categorical variables of GST and manual group.

Propensity score matching was performed to reduce the influence of confounding variables, controlling for age, BMI, smoking history, history of surgery, complications, and level of complexity of pneumonectomy.Patients were matched 1:1 by nearest neighbor-matching without replacement (caliper width, 0.03). The means of the outcomes were compared using t-test.

## Results

After conducting propensity score matching at 1:1 ratio for 186 patients at study entry, 108 patients were finally selected for analysis purposes, including 54 patients in the GST group and 54 patients in the manual group. Mean ages were 54.46 (SD = 10.64) and 55.19 (SD = 10.88) years old for the GST and manual group, respectively. There were no group-differences in the baseline demographic and clinical characteristics (Table 1).

**Table 1:** Baseline demographic variables and clinical information after propensity score matching (Total n = 108)

Characteristics	n (%) or mean ± SD		P-value
	GST (n = 54)	Manual (n = 54)	
Age	54.46 (10.64)	55.19 (10.88)	0.728
Height (cm)	160.46 ± 7.8	161.61 ± 6.99	0.422
Weight (kg)	58.53 ± 10.77	59.95 ± 9.9	0.475
BMI	22.67 ± 3.56	22.88 ± 2.99	0.737
Male	20 (37.0%)	18 (33.3%)	0.84
Smoking history			
Yes	11 (20.4%)	9 (16.7%)	0.804
Surgical history			
Yes	24 (44.4%)	28 (51.9%)	0.563
Surgical complexity			
Easy	25 (46.3%)	27 (50.0%)	0.896
Medium	15 (27.8%)	13 (24.1%)	
Hard	14 (25.9%)	14 (25.9%)	

*BMI* Body Mass Index

Table 2 shows the results of the clinical characteristics after propensity score matching. Diabetes (GST group: 11.1% vs manual group: 11.1%) and hypertension (GST group: 18.5% vs manual group: 22.2%) were the most common medical conditions for the two groups. The GST and manual groups did not significantly differ in cancer type and pulmonary function (p = 1.000 for both). In addition, the distribution of pleural adhesion and pulmonary fissures were similar between the GST and manual groups (p-values = 0.804 and 0.783, respectively). Moreover, tumor location, access type, and cancer stage were significantly different between the two groups (p-values = 0.082 and 0.155, respectively, see Table 2 for p-values for cancer stage).

**Table 2:** Clinical characteristics after propensity score matching

Events	n (%) or mean ± SD		p-value
	GST (n = 54)	Manual (n = 54)	
Cancer type			
Squamous cell carcinoma	1 (2.0%)	1 (2.0%)	1.000
Adenocarcinoma	42 (82.4%)	41 (80.4%)	
Other	8 (15.7%)	9 (17.6%)	
Tumor location			
Segment	34 63.0%)	24 (44.4%)	0.082
Lobe	20 (37.0%)	30 (55.6%)	
Access type			
Single port	20 (37.0%)	30 (55.6%)	0.155
Double port	17 (31.5%)	12 (22.2%)	
Triple port	17 (31.5%)	12 (22.2%)	
Pulmonary fissures			
Incomplete	16 (30.2%)	14 (25.9%)	0.783
Less incomplete	37 (69.8%)	40 (74.1%)	
Pulmonary fuction			
High	44 (81.5%)	44 (81.5%)	1.000
Moderate	10 (18.5%)	10 (18.5%)	
Pleural adhesions			
None	35 (64.8%)	36 (66.7%)	0.804
Mild	15 (27.8%)	16 (29.6%)	
Moderate	4 (7.4%)	2 (3.7%)	
Cancer stage			
TNM_T			
1	5 (14.7%)	3 (7.5%)	0.569
1a	18 (52.9%)	19 (47.5%)	
1b	8 (23.5%)	6 (15.0%)	
1c	0 (0%)	1 (2.5%)	
2	1 (2.9%)	3 (7.5%)	
2a	1 (2.9%)	3 (7.5%)	
2b	1 (2.9%)	2 (5.0%)	
3	0 (0%)	2 (5.0%)	
is	0 (0%)	1 (2.5%)	
TNM_N			
0	34 (100%)	37 (92.5%)	0.299
2	0 (0%)	3 (7.5%)	
TNM_M			
0	23 (67.6%)	32 (80.0%)	0.345
x	11 (32.4%)	8 (20.0%)	
Cormobidities			
Emphysema	2 (3.7%)	1 (1.9%)	1
Diabetes	6 (11.1%)	6 (11.1%)	1
Hypertension	10 (18.5%)	12 (22.2%)	0.811
Other	15 (27.8%)	10 (18.5%)	0.361

Table 3 shows the results of the primary clinical outcome measures. Compared with the manual group, GST group had significantly lower risks for both intraoperative bleeding (GST: 22.8% versus manual 51.9%, p = 0.003) and intraoperative intervention (GST: 31.5% versus manual: 55.6%, p = 0.02), controlling for age, gender, body mass index (BMI), smoking history, surgical history, medical conditions, and level of complexity of pulmonary resection. A decrease in the intraoperative blood loss was also observed in the GST group, but not statistically significant (GST: 134.39 ± 52.82 ml versus manual: 158.11 ± 73.14 ml, p = 0.102). The blood loss was measured by the fluid volumes (mostly blood) collected by the aspirator for the entire surgical procedure from thoracotomy to wound closure, therefore, the volumes of the blood loss measured in this way might be higher than the volumes measured by other methods such as weighting surgical gauze. In addition, Table 3 also shows that a higher proportion of patients required intraoperative intervention than that of patients with intraoperative bleeding. The reason for this phenomenon is that surgeons would evaluate the risk for air leakage and bleeding for patients with chronic obstructive pulmonary disease (COPD), emphysema, or suboptimal lung parenchyma quality after the pulmonary resection and determine whether a suture repair is needed to prevent future air leakage and bleeding.

**Table 3:** Primary intraoperative outcomes comparison between GST and manual group

Events	GST (n = 54)	Manual (n = 54)	p-value
Intraoperative bleeding			
Yes	12 (22.8%)	28 (51.9%)	0.003
Intraoperative intervention			
Yes	17 (31.5%)	30 (55.6%)	0.02
Intraoperative blood loss (ml)	134.39 ± 52.82	158.11 ± 73.14	0.102

Table 4 presents the analyses of the secondary clinical outcomes. The GST group had a significantly lower proportion of using NEOVEIL (GST: 24.1% versus non-GST: 50%, p = 0.01). No statistically significant differences were detected in intraoperative air leakage, conversion to open surgery, reoperation during the first admission, drainage tube placement (≥ 5 days), average operation time, and length of stay in the hospital.

**Table 4:** Secondary clinical outcomes comparison between GST and manual group

Events	n (%) or mean ± SD		p-value
	GST (n = 54)	Manual (n = 54)	
Neoveil usage	13 (24.1%)	27 (50.0%)	0.01
Intraoperative air leakage			
No bubble	48 (88.9%)	46 (85.2%)	0.556
Countable bubble	5 (9.3%)	8 (14.8%)	
Continuous bubble	1 (1.9%)	0 (0%)	
Drainage tube placement ≥ 5 days	6 (12.2%)	9 (19.6%)	0.486
Conversion to open surgery	0 (0%)	0 (0%)	1
Reoperatin during the admission	0 (0%)	1 (2.0%)	1
Operation time (mins)	118.04 ± 37.39	127.06 ± 46.22	0.281
LOS	7.78 ± 2.72	7.71 ± 2.38	0.877

*LOS* Length of stay

## Discussion

To our best knowledge, it was the first real-world study in China that demonstrated the effect of the GST stapling system on the perioperative outcomes of segmentectomy and lobectomy procedures. Our findings suggested that the GST group was associated with better intraoperative outcomes, compared to the manual group. The risk for intraoperative bleeding and intraoperative interventions was significantly reduced using the GST system. Among all the secondary clinical outcomes, the use of GST system is associated with lower NEOVEIL consumption. The two groups did not differ significantly in terms of drainage tube duration and average operation time, which could be attributed to the relatively small sample size.

Since the study was conducted within a Chinese hospital context, the research interests may differ from that of the previous studies that were conducted within other countries. However, our study still contributes to the knowledge of the real-world effectiveness of the GST system. Our study results resonated with the previous findings that the GST system and powered staplers were clinically superior to manual staplers. Rawlins et al. reported that the GST system lowered the risk for developing hemostasis-related complications during the laparoscopic sleeve surgeries (LSG) compared to the Signia™ stapling system, as the GST system was more powerful in reducing tissue movements [16]. The reduced tissue movements were likely to be attributed to that the GST system powered staplers can effectively reduce surgeons’ unwanted hand movements due to the superiority of the physical characteristics of powered staplers. Additionally, Fegelmen et al. observed that the use of GST system was associated with significantly fewer staple line interventions during LSG [18]. Furthermore, as aforementioned, Miller et al. observed that the use of powered stapler was associated with a lower risk of having hemostasis-related complications during the surgery and the powered stapler can also reduce the total length of stay in hospital [13]. Other researchers also reported comparable findings in terms of the clinical performance of powered staplers [15].

Apart from the clinical performance, the powered staplers also consumed fewer medical materials compared to the manual staplers. NEOVEIL, made of polyglycolic acid, is a bioabsorbable mesh sheet that has been used for surgical suturing or tissue strengthening [25]. One thing to note is that a higher consumption of NEOVEIL usually indicates a high incidence of intraoperative complications. In our study, we observed that the GST group was associated with significantly fewer consumption of NEOVEIL. Our finding is consistent with few studies have focused on the consumption of surgical supplies [19, 20]. For example, in a propensity score-matched study, Shigeeda et al. reported that the use of powered staplers was associated with less fibrin glue consumption [20] (fibrin glue is a biological adhesive that is used for hemostasis).

In addition, the surgeons’ choice of the staplers also played an important role in interpreting the results. This study is a non-interventional study, therefore, surgeons would choose the staplers based on their preference and the difficulty of the surgical procedures. In general, surgeons tend to use GST staplers for patients with COPD, pleural adhesion, emphysema, or deep-seated tumor. Therefore, even if we controlled for baseline characteristics such as the complications and the complexity of pneumonectomy, the proportion of complicated surgical procedures was still higher in the GST group (not statistically significant), which confirmed the better clinical performance of the GST stapler and partially explained why there were no significant differences in some of the secondary outcomes such as the length of stay.

This study has some limitations. One limitation is the absence of intraoperative leakage as a major outcome, given that no data were available on it and the study team cannot reliably recall the occurrence of this event. Secondly, The small sample size of this study prevent some comparisons from making statistical inference. In addition, given the nature of non-randomized study, we cannot randomize the stapler choice in the study design. Due to surgeons’ selection preference, there was a major selection bias for stapler choice in different surgical procedures and the study population was not representative as all the patients were from the single clinical site. For instance, surgeons chose to use GST power stapler in single port segmentectomy, and non-GST stapler in multi-port lobectomy. Owing to this selection of preference, we cannot compare the effectiveness of GST system between different procedures (i.e., single port segmentectomy vs multi-port lobectomy). Thus, the sample in this study had different surgical procedures (multi-port segmentectomy and single-port lobectomy). Ideally for this study, the effect of GST system would have been examined in comparable surgical procedures in order to eliminate the effect of surgeons’ difference/preference. Finally, information bias might occur due to the nature of retrospective study, although quality checks were implemented to reduce it. Moreover, a causal linkage could not be drawn between the use of GST system and better clinical outcomes as this study was an observational retrospective cohort study and so further prospective studies are needed.

## Conclusion

The use of GST system for pulmonary resection among lung cancer patients were associated with significantly lower risk for intraoperative bleeding, intraoperative interventions, and fewer NEOVEIL uses, compared with manual staplers. Future prospective clinical studies are needed to examine the clinical and economic outcomes on the use of the new stapling system.

## Data Availability

All authors understand that submission of a manuscript to a BMC journal implies that materials described in the manuscript, including all relevant raw data, will be freely available to any scientist wishing to use them for non-commercial purposes, without breaching participant confidentiality.

## References

[1] Fitzmaurice C, Abate D, Abbasi N, Abbastabar H, Abd-Allah F, Abdel-Rahman O (2019). Global, regional, and national cancer incidence, mortality, years of life lost, years lived with disability, and disability-adjusted life-years for 29 cancer groups, 1990 to 2017: a systematic analysis for the global burden of disease study. JAMA Oncol.

[2] Cao M, Chen W (2019). Epidemiology of lung cancer in China. Thorac Cancer.

[3] Parascandola M, Xiao L (2019). Tobacco and the lung cancer epidemic in China. Transl Lung Cancer Res.

[4] Liu C, Shi J, Wang H, Yan X, Wang L, Ren J (2021). Population-level economic burden of lung cancer in China: provisional prevalence-based estimations, 2017–2030. Chin J Cancer Res.

[5] Chen FF, Zhang D, Wang YL, Xiong B (2013). Video-assisted thoracoscopic surgery lobectomy versus open lobectomy in patients with clinical stage I non-small cell lung cancer: a meta-analysis. Eur J Surg Oncol.

[6] Bédat B, Abdelnour-Berchtold E, Perneger T, Licker MJ, Stefani A, Krull M (2019). Comparison of postoperative complications between segmentectomy and lobectomy by video-assisted thoracic surgery: a multicenter study. J Cardiothorac Surg.

[7] Liu L, Mei J, He J, Demmy TL, Gao S, Li S (2019). International expert consensus on the management of bleeding during VATS lung surgery. Ann Transl Med.

[8] Bédat B, Abdelnour-Berchtold E, Krueger T, Perentes JY, Ris HB, Triponez F (2018). Clinical outcome and risk factors for complications after pulmonary segmentectomy by video-assisted thoracoscopic surgery: results of an initial experience. J Thorac Dis.

[9] Tsunezuka Y, Tanaka N, Fujimori H (2020). The impact of endoscopic stapler selection on bleeding at the vascular stump in pulmonary artery transection. Med Devices (Auckl).

[10] Acuff TE, Mack MJ, Landreneau RJ, Hazelrigg SR (1993). Role of mechanical stapling devices in thoracoscopic pulmonary resection. Ann Thorac Surg.

[11] Özyurtkan MO, Kaba E, Toker A (2017). Technological innovation in video-assisted thoracic surgery. J Vis Surg.

[12] Kuroda H, Yoshida T, Sakao Y (2017). A powered vascular staple for the application of segmental bronchial closure in thoracoscopic anatomic segmentectomy. J Thorac Dis.

[13] Miller DL, Roy S, Kassis ES, Yadalam S, Ramisetti S, Johnston SS (2018). Impact of powered and tissue-specific endoscopic stapling technology on clinical and economic outcomes of video-assisted thoracic surgery lobectomy procedures: a retrospective, observational study. Adv Ther.

[14] Roy S, Yoo A, Yadalam S, Fegelman EJ, Kalsekar I, Johnston SS (2017). Comparison of economic and clinical outcomes between patients undergoing laparoscopic bariatric surgery with powered versus manual endoscopic surgical staplers. J Med Econ.

[15] Park SY, Kim DJ, Mo Nam C, Park G, Byun G, Park H (2019). Clinical and economic benefits associated with the use of powered and tissue-specific endoscopic staplers among the patients undergoing thoracoscopic lobectomy for lung cancer. J Med Econ.

[16] Fegelman E, Knippenberg S, Schwiers M, Stefanidis D, Gersin KS, Scott JD (2017). Evaluation of a powered stapler system with gripping surface technology on surgical interventions required during laparoscopic sleeve gastrectomy. J Laparoendosc Adv Surg Tech.

[17] Kimura M, Tanaka H, Hato M, Taniwaki S, Shibata Y, Mizuno K (2016). Evaluation of a new stapler with unique surface gripping technology. Br J Med Med Res.

[18] Rawlins L, Johnson BH, Johnston SS, Elangovanraaj N, Bhandari M, Cohen RV (2020). Comparative effectiveness assessment of two powered surgical stapling platforms in laparoscopic sleeve gastrectomy: a retrospective matched study. Med Devices (Auckl).

[19] Murata M, Umeda M, Takeuchi J, Suzuki H, Shibuya Y, Shigeta T (2011). Application of polyglycolic acid sheet (Neoveil^®^) and fibrin glue spray (Bolheal^®^) for open wounds in oral surgery. J Jpn Stomatol Soc.

[20] Shigeeda W, Deguchi H, Tomoyasu M, Kudo S, Kaneko Y, Kanno H (2021). Utility of the powered stapler for radical pulmonary resection: a propensity score-matched analysis. Surg Today.

